# Public Health Education in South Asia: A Basis for Structuring a Master Degree Course

**DOI:** 10.3389/fpubh.2014.00088

**Published:** 2014-07-21

**Authors:** Rajendra Karkee

**Affiliations:** ^1^School of Public Health and Community Medicine, B. P. Koirala Institute of Health Sciences, Dharan, Nepal

**Keywords:** public health education, postgraduate course, South Asia, public health practice, public health workforce

## Abstract

Countries in South Asian Association for Regional Cooperation (SAARC) lack enough public health workforces to address their poor public health situation. Recently, there have been efforts to develop capacity building in public health in these countries by producing competent public health workforce through public health institutes and schools. Considering the wide nature of public health, the public health education and curricula should be linked with skills, knowledge, and competencies needed for public health practice and professionalism. The 3 domains of public health practice and the 10 essential public health services provide an operational framework to explore this link between public health practice and public health education. This framework incorporates five core areas of public health education. A master degree course in public health can be structured by incorporating these core areas as basic and reinforcing one of these areas as an elective followed by a dissertation work.

## Introduction

Health is an important determinant of economic prosperity and development of a nation (1), and central to the achievement of all millenium development goals (2). One way to improve health is to build up public health capacity by strengthening the public health education and by producing public health workforce (3), since the empirical evidence suggests that major improvement of health does not come from new medical findings or cures, but from the broad development and application of population-based preventive programs (4).

The South Asian countries under the South Asian Association for Regional Cooperation (SAARC) include Afghanistan, Bangladesh, Bhutan, India, Maldives, Nepal, Pakistan, and Sri Lanka. Excluding Maldives and Sri Lanka, the remaining six countries have nearly one-fifth of the world’s population with about 27% of global disease burden (680,859 thousands disability-adjusted life years lost in 2010) (5). These countries are undergoing an epidemiological transition with a double burden of diseases, unfinished agenda of infectious diseases, nutritional deficiencies, and unsafe pregnancies, as well as the challenge of escalating epidemics of non-communicable diseases (6). Despite this situation, public health education in these countries has largely been neglected compared with medical education until recently, resulting in inadequate public health schools and workforce (3, 7).

WHO Regional Office for South-East Asia has called for a paradigm shift in approach to public health by focusing on a preventive and promotive health system that can actively change conditions that make people sick and by producing a public health workforce through public health institutes and schools in its landmark conference in Calcutta (8, 9). As a result, efforts have been already made to establish new public health institutes, schools, and public health education networking in these countries (6). For example, Public Health Foundation of India has been working to establish several Indian institutes of public health since 2006 and BP Koirala Institute of Health Sciences in Nepal started a School of Public Health in 2005 (7, 10).

## Nature of Public Health

It has been noted that health is determined not only by individual behavioral factors but also by population related social, economic, political, cultural, and environmental factors (11). In fact, public health evolved from narrow disease-focused to broader population-based, multidisciplinary, and multisectoral disciplines as “collective action for sustained population-wide health improvement” (12). It needs contributions from various disciplines: sociologist, economist, politician, environmentalist, epidemiologist, statistician, clinician, etc. It needs to collaborate with different sectors: education, nutrition and food production, employment and income generating activities, water and sanitation, housing, etc. This means that public health is everybody’s business and responsibility, achievable only if health in a broad sense becomes a central concern of the policy-making process with prime role played by the state. Such wide nature of public health might pose a challenge to organizing public health education and developing capacity building when compared to medicine or nursing education (13). In fact, there is a great variation in institutes and courses offered in the South Asian countries (9). A post-graduate course needs to incorporate intersectorial, interdisciplinary, and community-oriented nature of public health. A framework of post-graduate course provision can guide the course development (14).

## The Three Domains and 10 Activities of Public Health

**Figure 1:**
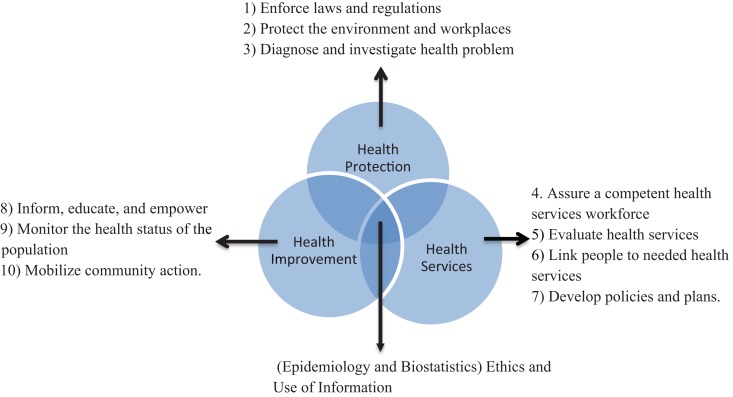
**The 3 domains and 10 activities of public health practice**. Modified from Ref. (15).

A framework consisting of three domains of public health is useful to manage the wide nature of public health and to clarify its boundaries in terms of public health practice and public health education (15). The three domains, health improvement, health protection, and health services, are inter-related with a common core (Figure 1). The common core includes research methods (epidemiology and biostatistics), ethics, and use of information. Health improvement domain includes socio-economic influences and health promotion, tackling the underlying determinants of health; health protection domain includes infectious diseases control, disaster prevention, environmental health regulation, and occupational health; and health services domain includes health care system and policy, service quality, health care management, evidence-based practice, and health economics. The 10 essential public health services have been identified (16), which can be divided among the three domains (Figure 1).

The 10 public health services indicate the settings where public health professionals work. Thus, the framework can also guide to outline the basic competencies required for public health graduates. It can help to discover the interface between public health education and public health practice, thereby showing how the public health education can be organized to support the development of competent public health workforce to deliver public health programs. It can be utilized to construct a basic competency-based model in post-graduate education in public health to achieve 10 essential public health services. Building on this basic model, detail competencies need to be specified in chosen concentration area and be tailored to the individual need of a school or institute so that the post-graduate program is effective and relevant to its context. Competencies for public health course have been formulated in different countries: by the council on linkages between Academia and Public Health Practice (17); by Association of School of Public Health in USA (18); by public health Agency of Canada (19); and by the Association of Schools of Public Health in the European Region (20). Similarly, competencies for Master of Public Health course in Australia, India, and in other low and middle-income countries have been prepared (14, 21, 22).

## Basis of Structuring a Post-Graduate Public Health Course

A post-graduate course in public health can start with core courses representing all the three domains, with an opportunity to gain advanced knowledge in one of those core courses. This can be fulfilled by structuring the post-graduate course in three parts: core course, electives, and thesis.

### Core course

The core course is not considered as in depth study but basic knowledge and skills needed to perform all public health services in all the three domains. The core course should include basic knowledge in these areas: epidemiology; biostatistics; health policy, management, and economics (health services administration); social and behavioral sciences (medical sociology, health education, health promotion, behavior change); and environmental health. These are internationally agreed five core areas of public health, which are consistent with the three domains of public health practice, and with curriculum proposed by the Associations of School of Public Health in USA and Europe (18, 20); and competencies based course design for Public Health in Australia (22). The core course also needs to incorporate the globalization impact on health as globalization has been acknowledged as one of the determinants of health in this era of interdependence (23). Besides, urbanization, population aging, and health disparities are new challenges in the twenty-first century, and hence, worthy of inclusion in core component. These new challenges will largely shape the national and global health (24).

In addition to these core areas, public health ethics and public health skill development (writing and speaking skills, management and leadership skills, conflict management, interpersonal relationship, negotiation skills with politicians and media, literature search, computer skills) should also be added to the basic component. These skills have been increasingly recognized as crucial for effective public health practice (18, 25). Especially, a recent Lancet report on education of health professionals for the twenty-first century has made three recommendations for future health education that include public health skill development: informative and transformative learning that bridges the gap between science and practice; training in teamwork and interpersonal relationships; and technology (computers and communication) (13).

### Elective Courses

Owing to broad nature of public health, it is desirable to gain in depth knowledge in a particular concentration area. A wide variety of elective courses and modules can be offered in each domain (Table 1). The elective courses can focus on particular population groups, for example, maternal and child health, mental health, etc. Elective courses should be guided by what type of health professionals the community or the country needs more, what the most unmet health needs of the communities are, and what employment opportunities for the graduates exist. Incorporation of practical field-based studies should be encouraged and included because the public health skills and management are best learnt in the field. Such community based field studies can be conducted as frequent small mini projects, field site observations in parallel with classroom teaching or as an internship in the form of real public health practice by placing students in relevant community organizations, government health departments, or non-government organizations.

**Table 1:** **Examples of elective modules in each domain of public health practice**.

Health improvement	Health protection	Health services
Primary health care Public health nutrition	Advanced epidemiology and biostatistics	Health care policy and management
	∙Design and analysis of epidemiological studies	∙Health leadership and management
∙Maternal and child nutrition	∙Statistical methods in epidemiology	∙District health management
∙Nutrition policy and programing	∙Designing disease control programs	∙Health systems design and management
∙Nutrition assessment and malnutrition	∙Infectious diseases epidemiology	∙Health system research
Medical sociology and anthropology	∙Non-communicable diseases epidemiology	Maternal and child health
Health promotion	AIDS	∙Integrated management of childhood illness
Health education and behavior change	Disaster and post-disaster management	∙Safe motherhood and prenatal health
	Environmental health	Reproductive/sexual health
	∙Medical entomology	∙Family planning programs
	∙Hygiene, water, and sanitation interventions	Health economics
	Occupational health	∙Health care financing
	Globalization and health	∙Health sector reform and financing
		∙Health care evaluation
		Hospital administration

### Thesis

The master research project is an opportunity for a student to deal with a public health issue, commonly in his/her concentration area. It demands integration of empirical data, theory, and methods. Students are familiarized with data collection, study design, literature search, interpretation of data, and independent scientific writing. It is desirable that thesis be based on primary data obtained from fieldwork but thesis can also be based on secondary data, desk-study in the form of systematic review, metaanalysis, or other project work.

## Conclusion

The multidisciplinary and multisectorial nature of public health might pose a challenge to organizing public health education. The three domains of public health practice provide an operational framework to explore the interface between public health practice and public health education. This framework incorporates five core areas of public health education. A post-graduate course in public health can be structured by incorporating the five core areas of public health as basic subjects and reinforcing one of those subjects as an elective followed by a dissertation work.

## Conflict of Interest Statement

The author declares that the research was conducted in the absence of any commercial or financial relationships that could be construed as a potential conflict of interest.
